# Specialization in Dentistry

**Published:** 1919-09

**Authors:** 


### SPECIALIZATION IN DENTISTRY

The development of dentistry within the past decade has been so rapid that it has come to be looked upon as beyond the ability of the average practitioner to encompass singly the practice of the profession in all its branches. The increased demand for dentistry in general and for a higher class of work in particular as the result of the enlightenment of the public on subjects dental has stimulated the profession to devise ways and means of meeting the demand for higher-class work, and it seems that the most expeditious method of meeting this demand is by increased efficiency in the different branches, with a logically increased efficiency for the whole—hence the day of specialists in dentistry. Specialization is in the dental air, and within the past few years it has received an impetus which threatens to carry us to a point where it may lessen to a degree the general humanitarian value of dental service to the public.

The oral hygiene propaganda has resulted in a material increase in the demand for dentistry, and specialization will certainly not be a means of meeting this increased demand at a time when the profession is already taxed far beyond its capacity. Specialization may meet the demand for better dentistry, but what is to become of the masses of people, the people we are endeavoring to educate, and with a large degree of success, as to the value of dentistry, the class that needs it most? They certainly can not afford the services of the various specialists, and dentistry, we dare say, will become more of a luxury to this class than it is at the present time.

The narrowing influence of specialization will, we fear, tend to emphasize more pronouncedly the criticism which has already been raised against dentistry as a specialty of medicine, namely, that the dental specialist inevitably comes to view all diseased conditions of the human body through the narrow lenses of his particular specialty, and therein we believe may lie a source of detriment to dentistry. Dentistry at best is more or less narrowing, and the continued subdivision of a specialty into sub-specialties will in the nature of the case exert more strongly this narrowing influence. The average dentist in the past has been charged, and with a degree of justification, with inability to see beyond the tooth, and if our specialization tendency leads us, as it now threatens to do, to the point where some will eventually confine their efforts to a particular portion of the tooth, then the logical eventuality will be that the sub-specialist will be charged with not being able to see beyond his particular portion of the tooth. In the light of the foregoing premise is it not appalling to contemplate the restricted mental vision which may possibly result from confining one's activities solely to one portion of the tooth or one limited phase of dentistry, and is there not the danger of developing what President Wilson has termed a "one-track mind," with too narrow a track?

The development of specialties will doubtless enable the specialist to demand higher fees, which everyone agrees would be helpful to the profession on the whole; but something more than the desire for higher fees should be the stimulus which eventuates in the announcement of a dentist as having confined his practice to a particular specialty of dentistry. In speaking of this phase of specialization in dentistry, Dr. Paul Stillman, in his report on "Practice" before the Dental Society of the State of New York, very aptly says:

"The entering into specialization signifies a frankly acknowledged ability in some chosen special branch. When an announcement is backed by real ability plus experience, the world is better for the change, perhaps, but when a specialist is announced whose only excuse is a greedy desire to profit at the expense of the profession and the public and a desire for larger fees and shorter hours, every man in the profession is at once personally injured."

There is, however, much to be said on the other side of the question. It is only logical to assume that the dentist who specializes in one phase of dentistry will become more proficient in that phase than one who undertakes all phases of general practice, provided the specialty is the mature development of long experience in the general practice of dentistry. We readily admit that, other things being equal, the specialist should be able to render more efficient service in his particular branch than the general all-round man, and this is forcibly exemplified in specialization within the medical profession. We have also an exemplification of the narrowing influence of specialization in our sister profession. Who has not heard and known of the surgeon who has specialized in one line of work, diagnosing and operating for almost all conditions in the belief that he is treating the condition with which he is most familiar? For instance, to what ridiculous extent has not the appendicitis fad carried specialists who are prone to see an inflamed appendix in almost every abdominal cavity that exhibits symptoms of disease!

But we need not go to medicine or surgery for examples of fetishism. In our own profession we are all only too familiar with the eagerness with which the dental profession seizes upon a new method of procedure. We are today in the midst of a wave of enthusiastic condemnation of septic teeth as the cause of systemic conditions. We have over-enthused over the use of the X-ray as a diagnostic agent which shows us the evils of past practice, which is causing many of our leading operators to reverse their opinions of tooth conditions as well as their methods of procedure. We do not deprecate the value of the X-ray as a diagnostic aid, nor do we wish to minimize the dangerous potentialties of oral focal infections, but simply cite these as instances of our readiness as a profession to adopt and carry to extremes new methods and procedures. One of our leading advocates of the use of the X-ray in dentistry, as the result of observing the ability of the novitiate in X-ray work to see almost anything he is looking for in the X-ray film, sounds this timely note of warning:

"In order to make a good diagnosis we must not become obsessed with the infallibility of any particular diagnostic agent, because the wider and longer experience a man has, the greater will be the caution with which he relies upon any one agent."

Some of the leading members of our profession have become so, shall we say, obsessed with the idea and have so ardently advocated the subdivision of dentistry, that we now have the following distinct specialties in dentistry: Oral Surgery, Prosthodontia, Exodontia, Orthodontia,

Periodontia, Radiodontia, and more recently recognized as such, specialists in Crown and Bridge Work—or shall it be Pontiodontia?—specialists in root-canal work, apicoectomy, etc.

Shades of Webb, Varney, Perry, *et al!* What has become of operative dentistry, the foundation upon which modern dentistry was built, and we dare say the most important, the most difficult, and the most neglected branch of our profession? True, the ultimate aim of our present oral hygiene propaganda is the elimination of operative reparative procedures as well as all other reparative procedures, but we do not believe the operative dentist should be eliminated before we eliminate the need of his services.

As an ardent advocate of specialization in dentistry our esteemed contemporary, the editor of the *Dental Items of Interest,* is a conspicuous example, and so strongly does he believe in the cause that he has recently undertaken almost single-handed a propaganda for the establishment of a definite and distinct specialty in bridge work, and to be more specific, removable bridge work. In the light of our present knowledge of focal infections and oral sepsis we are not sure but what he has exhibited an unusual degree of perspicacity in advocating removable bridge work specifically, for if the efforts of some medical and dental enthusiasts in the direction of eliminating all suspected oral foci of infection are successful, all bridge work will be "removed" from the mouth and consigned to the scrap-heap in the future. However, we believe that day to be as far remote as the day when we shall see the elimination of the need for operative dentistry.

We believe that the future of bridge work, and more particularly removable bridge work, will be largely and almost entirely dependent upon the final outcome of the present research being done in regard to the relation of devitalized teeth as septic foci to serious systemic conditions. One of our contributors in the present issue goes so far as to say that "Every devitalized tooth is a septic tooth." What, then, is to become of removable bridge work, with its necessary devitalization of teeth for use as abutments? We are not unmindful of the fact that bridges in some, but we believe comparatively few, instances can be placed on vital teeth, but the great bulk of removable bridge work must in the nature of the case be placed on pulpless teeth. In the meantime, many of our expert bridge workers will continue to perform good service with both removable and fixed bridge work, for we believe the success of bridge work lies not so much in the question of whether it is fixed or removable as it does in the manner in which it is constructed, and beyond all other considerations the foundations upon which it is built.

We welcome the practice of specialists in dentistry in so far as they will increase the efficiency of individual efforts, but let us not succumb to the evil surrounding most specialties, that of making the balance of practice subordinate and of secondary importance to the specialty itself. Let us not forget the ideals of modern dentistry, and keep in mind the fact that a given specialty is only a contributing factor to the ultimate aim of modern medicine—prevention.—Editorial in August *Cosmos*.

